# A CALB-like Cold-Active Lipolytic Enzyme from *Pseudonocardia antarctica*: Expression, Biochemical Characterization, and AlphaFold-Guided Dynamics

**DOI:** 10.3390/md23120480

**Published:** 2025-12-15

**Authors:** Lixiao Liu, Hackwon Do, Jong-Oh Kim, Jun Hyuck Lee, Hak Jun Kim

**Affiliations:** 1 Department of Chemistry, Pukyong National University, Busan 48513, Republic of Korea; liulixiao@pukyong.ac.kr; 2 Division of Life Sciences, Korea Polar Research Institute, Incheon 21990, Republic of Korea; hackwondo@kopri.re.kr (H.D.); junhyucklee@kopri.re.kr (J.H.L.); 3 Department of Microbiology, Pukyong National University, Busan 48513, Republic of Korea; jokim@pknu.ac.kr

**Keywords:** cold-active lipolytic enzyme, *Pseudonocardia antarctica*, catalytic triad, cold-adaptation

## Abstract

Cold-active lipolytic enzymes enable low-temperature biocatalysis, but remain underexplored in Antarctic actinomycetes. Here, we report the discovery and first-step characterization of a CALB-like cold-active lipolytic enzyme (PanLip) from *Pseudonocardia antarctica*. Sequence and structure analyses revealed a canonical α/β-hydrolase fold with a conserved Ser–Asp–His triad and short helical elements around the pocket reminiscent of CALB’s α5/α10 lid. Mature PanLip was expressed primarily as inclusion bodies in *E. coli*; an N-terminally truncation (PanLipΔN) improved solubility and PanLipΔN was purified by Ni–NTA. Far-UV CD confirmed a folded α/β architecture. PanLipΔN favored short-chain substrates (*p*-NPA, *k*_cat_/*K*_M_ = 2.4 × 10^5^ M^−1^·s^−1^) but also showed measurable hydrolytic activity toward natural triglycerides, consistently with a lipase-family esterase. The enzyme showed an activity optimum near 25 °C and pH 8.0. The enzyme tolerated low salt (maximal at 0.1 M NaCl), mild glycerol, and selected organic solvents (notably n-hexane), but was inhibited by high salt, Triton X-100, and SDS. AlphaFold predicted high local confidence for the catalytic core; DALI placed PanLip closest to fungal lipases (AFLB/CALB). Temperature-series MD and CABS-flex indicated enhanced surface breathing and flexible segments adjacent to the active site—including a region topologically matching CALB α10—supporting a flexibility-assisted access mechanism at low temperature. Structure-based MSAs did not support a cold adaptation role for the reported VDLPGRS motif. Taken together, these findings position PanLip as a promising cold-active catalyst with CALB-like access control and potential for low-temperature biocatalysis.

## 1. Introduction

Lipolytic enzymes (EC 3.1.1.x), including lipases and esterases, constitute a major group of the α/β-hydrolase superfamily and catalyze the hydrolysis of ester bonds in a broad range of substrates [1,2]. Due to their broad substrate specificity, regioselectivity, and ability to function under mild reaction conditions, lipolytic enzymes have been extensively utilized in various biotechnological and industrial fields, including organic synthesis, food and dairy industries, pharmaceutical manufacturing, biofuel production, and detergent formulations [3,4,5]. Among these, cold-active lipolytic enzymes, in particular, derived from psychrophilic microorganisms, have attracted significant attention because of their high catalytic efficiency at lower temperatures [6,7,8,9]. This feature provides substantial advantages in reducing energy consumption, preserving the activity of heat-sensitive substrates, and minimizing undesirable side reactions.

Psychrophilic microorganisms produce enzymes adapted to cold environments, characterized by higher catalytic activity at lower temperatures and typically reduced stability at elevated temperatures [10,11,12,13,14]. This thermal adaptability is largely attributed to increased structural flexibility in their active sites, allowing effective catalysis even under conditions of low kinetic energy [15,16,17]. Cold-active lipolytic enzymes have been characterized from diverse microorganisms inhabiting cold environments, such as polar regions, glaciers, and deep-sea ecosystems [7,18,19,20,21,22,23,24,25,26]. However, despite these advancements, the current repertoire of cold-active lipolytic enzymes remains limited, particularly those from Antarctic actinomycetes, which represent a relatively unexplored group. Actinomycetes from extreme Antarctic ecosystems exhibit unique biochemical and molecular adaptations, making them promising sources for novel enzyme discovery [27,28,29].

*Pseudonocardia antarctica*, isolated from the McMurdo Dry Valleys, Antarctica, represents one such actinomycete adapted to cold environments [30]. *Pseudonocardia*, a genus of actinobacteria, has received little attention regarding lipase characterization, especially those isolated from extreme Antarctic environments. Investigating enzymes from such environments can uncover novel enzymatic properties and catalytic mechanisms that could be invaluable for industrial processes. Hence, the detailed characterization of lipases from Antarctic *Pseudonocardia* species presents a significant research opportunity to discover enzymes with unique structural and catalytic traits suitable for industrial applications.

Although cold-active lipolytic enzymes are widely studied, enzymes from Antarctic genus *Pseudonocardia* remain largely unexplored, representing a significant gap in current enzyme biotechnology research. In this context, the present study aims to characterize a novel cold-active lipolytic enzyme derived from *P. antarctica*. Preliminary sequence analyses have indicated significant similarity between this enzyme and lipase B enzymes from *Aspergillus fumigatus* [31] and *Candida antarctica* [32], both extensively studied due to their robust industrial applications. Given these promising initial findings, this study focuses on cloning, expression, purification and comprehensive bioinformatic characterization of cold-active lipolytic enzyme from *P. antarctica*. Further, functional characterization assays were conducted to assess its biochemical properties, along with simple molecular dynamics analyses to gain preliminary insights into the structural basis underlying its activity at low temperatures. By exploring this novel enzyme’s enzymatic profile, this study provides critical insights into its potential suitability for biotechnological applications, particularly in processes operating at low temperatures. These findings not only address a critical research gap but also offer valuable theoretical and practical guidance for future enzyme engineering and industrial applications of psychrophilic lipases.

## 2. Results and Discussion

### 2.1. Primary Structure Analysis and Classification of PanLip

*P. antarctica* is an aerobic, Gram-positive actinobacterium first isolated from the extreme cold desert environment of the McMurdo Dry Valleys in Antarctica [30]. The origin of this microorganism strongly suggests that its lipolytic enzyme, designated as PanLip, would be adapted for function at low temperatures. The primary structure analysis revealed that the putative lipase PanLip (UniProt ID: A0A852WCG0) consists of 324 amino acids and contains an N-terminal signal peptide spanning the first 30 residues (Figure S1a). Signal peptide prediction further indicated that this region corresponds to a Sec-type signal peptide, suggesting that PanLip is secreted via the Sec pathway in its native host. Excluding this signal sequence, the mature protein has a calculated molecular weight of approximately 36.8 kDa. Interestingly, immediately downstream of the signal peptide, PanLip contains a ca. 40 residue-long proline-rich N-terminal extension that is likely to be a putative propeptide (Figure S1b). Similar N-terminal propeptides have been identified in fungal and bacterial lipases [33,34,35,36,37] and are known to act as intramolecular chaperones [38] or regulators of the catalytic efficiency and substrate selectivity [39]. Presumably, the putative propeptide of PanLip plays a similar role, although its function remains to be experimentally validated.

Since cold adaptation is frequently assessed from the primary sequences of cold-active proteins [15,16,40,41], PanLip’s primary amino acid composition analyzed with a focus on properties commonly associated with cold adaptation features. These include increased structural flexibility via lower proportions of rigidifying residues (e.g., arginine, proline), altered charge distribution, and reduced hydrophobicity. However, the PanLip sequence contains a relatively high percentage of proline (10.4%) and arginine (4.3%), and an exceptionally low lysine content (0.3%), resulting in a Lys/Arg ratio of 0.067. This is markedly lower than values typically observed in psychrophilic enzymes [15,42,43]. Additionally, PanLip exhibits a moderate aliphatic index (79.91) and a slightly negative GRAVY score (–0.120), indicating an overall moderate thermostability and slight hydrophilicity. Collectively, while the protein does exhibit some cold-adaptive trends (e.g., modest hydrophilicity), its unusual residue distribution—particularly the arginine dominance and lysine scarcity—suggests that it may not conform to canonical models of cold adaptation. Instead, PanLip like other cold active proteins, may rely on alternative mechanisms such as local flexibility hotspots, structural dynamics, or surface charge modulation for maintaining activity at low temperatures [17,41,44,45].

**Figure 1:**
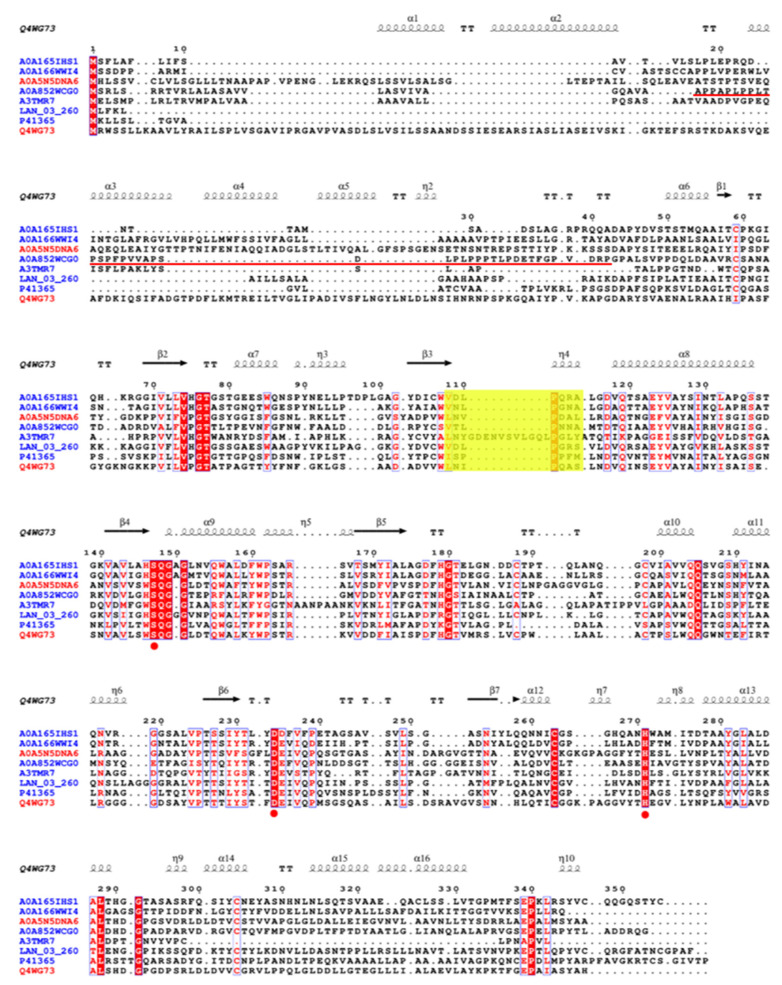
Multiple sequence alignment (MSA) of lipases from various organisms aligned using T-Coffee Expresso based on 3D structural information. The alignment includes two mesophilic lipases, *Lasiodiplodia theobromae* (A0A5N5DNA6, PDB ID: 7V6D) and *Aspergillus fumigatus* (Q4WG73, PDB ID: 6IDY), and six psychrophilic or psychrotolerant lipases from *Glaciozyma antarctica* PI12 (LAN_03_260), *Pseudonocardia antarctica* (A0A852WCG0), *Janibacter* sp. HTCC2649 (A3TMR7, PDB ID: 7V3K), *Calocera cornea* (A0A165IHS1), *Athelia psychrophila* (A0A166WWI4), and *Candida antarctica* lipase B (P41365, PDB ID: 4K6G). The accession numbers for mesophilic and psychrophilic lipases were written in red and blue, respectively. Secondary structure annotations are based on the *A. fumigatus* lipase (6IDY). The conserved catalytic triad residues (Ser169, Asp253, His292) are indicated with red circles below the aligned sequences. The cold-adaptation motif VDLPGRS proposed based on the structural analysis of lipase from *G. antarctica* PI12 (LAN_03_260) is highlighted in a yellow box. The putative propeptide region of PanLip is underlined in red. The figure was generated using ESPript 3.0 (https://espript.ibcp.fr/ESPript/ESPript/, accessed on 10 March 2025).

Multiple sequence alignment (MSA) of PanLip with psychrophilic and mesophilic lipases of highest identities was performed to identify conserved residues, infer evolutionary relationships, and reveal functionally or structurally important regions among homologous proteins. The PanLip (A0A852WCG0) exhibited moderate sequence identities with both mesophilic and psychrophilic fungal and bacterial lipases (Figure 1). It showed ~30% identity with the *Candida antarctica* lipase B (P41365, PDB ID: 4K6G), *Calocera cornea* (A0A165IHS1), and *Glaciozyma antarctica* PI12 lipase (LAN_03_260), ~29% with *Athelia psychrophila* (A0A166WWI4), and 25% with *Janibacter* sp. HTCC2649 lipase (A3TMR7, PDB ID: 7V3K). In comparison, it shared 29% identity with the mesophilic *Aspergillus fumigatus lipase* (Q4WG73, PDB ID: 6IDY) and 27% with the *Lasiodiplodia theobromae* lipase (A0A5N5DNA6, PDB ID: 7V6D). A motif, VDLPGRS, from *G. antarctica* lipase (LAN_03_260), was recently proposed as potentially essential for cold adaptation, contributing to structural stability and function at low temperatures [42]. However, the MSA analysis presented here, based on lipase sequences from various organisms, challenges the uniqueness and specificity of the VDLPGRS motif (Figure 1 and Figure S2). The alignment reveals substantial conservation of the VDLPGRS region across lipases from both psychrophilic and mesophilic actinomyctes, suggesting that this motif may not exclusively define psychrophilic adaptation. Specifically, mesophilic lipases from *L. theobromae* and *A. fumigatus* exhibit high sequence similarity within this motif, arguing that the VDLPGRS sequence is solely critical for psychrophilic stability and catalytic activity.

**Figure 2:**
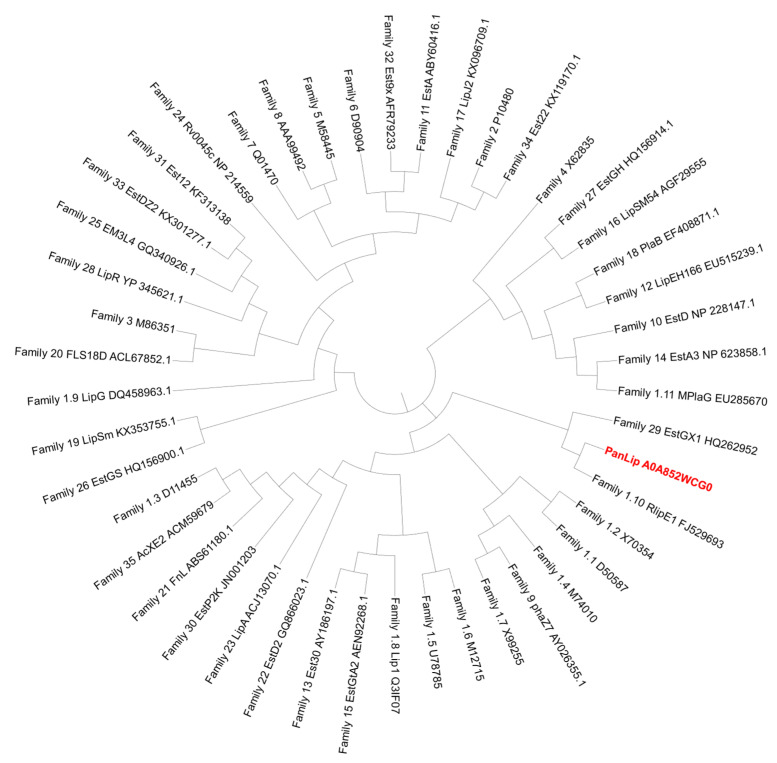
Phylogenetic tree of 35 representative lipolytic enzyme families constructed using MEGA 12.0.11. Each branch represents one UniProt--annotated sequence per family. *Pseudonocardia antarctica* lipase (PanLip) is highlighted in bold red and clusters most closely with Family I.10. The tree was inferred by the Maximum-Likelihood method with the Jones–Taylor–Thornton (1992) substitution model.

To classify lipolytic enzyme family of PanLip among known bacterial lipolytic enzyme families, a phylogenetic tree (Figure 2) was constructed following the classification criteria established by Hitch and Clavel (2019) [46]. In this classification, enzymes exhibiting ≥60% sequence identity are considered to belong to the same lipase family, a threshold that represents three standard deviations above the mean inter-family similarity (30.9 ± 9.6%). In the pairwise analysis, PanLip did not display sequence identity exceeding 60% with any representative member from the 35 previously defined lipolytic enzyme families (as shown in Table S1). Although PanLip has catalytic serine within the pentapeptide GHSQG motif as in family XIX, it shows 37% similarity to this family [47]. This finding suggests that PanLip likely represents a distinct or divergent member within the bacterial lipase superfamily, potentially forming a novel subfamily related to family I.10, as indicated by its clustering pattern in the phylogenetic tree (Figure 2). The relatively low sequence identity, together with its unique amino acid composition and conserved catalytic triad, implies that PanLip may have evolved specialized structural adaptations distinct from canonical lipase families.

### 2.2. Cloning, Expression, and Purification of PanLip Proteins

**Figure 3:**
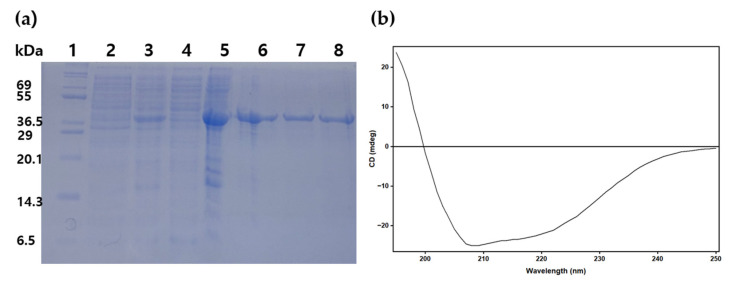
SDS-PAGE (**a**) and far-UV circular dichroism (CD) spectrum (**b**) of PanLipΔN. (**a**) The N-terminal 27 residue-deleted PanLip (PanLipΔN) and PanLipΔN S169A were cloned in pET28 vector, expressed in SHuffle strain with 0.1 mM IPTG, and purified using Ni-NTA affinity chromatography. Lane 1, protein molecular marker (K08000, KOMA biotechnology, Seoul, Republic of Korea); lane 2, uninduced total cell; lane 3, induced total cell; lane4, supernatant of cell lysate; lane 5, insoluble fraction of cell lysate; lane 6, elution of PanLipΔN with 150 mM imidazole; lane 7, elution of PanLipΔN with 200–250 mM imidazole; lane 8, purified PanLipΔN S169A. (**b**) A 200 μg·mL^−1^ of PanLipΔN was prepared in 10 mM sodium phosphate buffer pH 7.4 and placed in a 0.1 cm path-length quartz cuvette. The far-UV CD was measured over 190–250 nm at 25 °C.

The signal peptide-deleted mature PanLip was expressed from pET-22b(+), pET-28a(+), and pET-32a(+) in both *E. coli* BL21(DE3) and SHuffle strains and observed predominantly as inclusion bodies. The expression in pET-22b(+) was very low, while in pET-28a(+) and pET-32a(+) were mild. However, all expressions were remained insoluble. In our expression trials, the full-length PanLip containing its native signal peptide was not evaluated. Notably, actinomycete-derived signal peptides, such as acetyl xylan esterase A of *Streptomyces lividans* [48] and xylanase from *Kocuria* sp. 3-3 [48,49], have successfully facilitated extracellular secretion in *E. coli*. Therefore, it is plausible that the native PanLip signal peptide could similarly support soluble or secretory expression, representing a promising avenue for further investigation. To reduce inclusion body formation, we generated PanLipΔN construct by removing the proline-rich 27 residues from the N-terminus that are predicted with low confidence in PanLip’s 3D structural model. These residues constitute the putative propeptide region of PanLip. In addition, PanLip contains six cysteine residues, and the AlphaFold model predicts the formation of three disulfide bonds (Cys87–Cys129, Cys210–Cys216, and Cys284–Cys326) within the folded protein, as discussed in the structural analysis below. To facilitate proper oxidative folding during heterologous expression, PanLipΔN was produced in the SHuffle T7 Express strain, which enables cytoplasmic disulfide bond formation. Expression of PanLipΔN using pET-28a(+) at low temperature (0.1 mM IPTG, 20 °C, 16 h) resulted in markedly improved solubility relative to the mature construct, although a substantial portion (>70%, visual estimate) remained in the insoluble fraction (Figure 3) [50,51]. Soluble PanLipΔN was purified by Ni^2+^–NTA with stepwise imidazole elution (200–250 mM) to homogeneity, yielding 0.3 mg·L^−1^ culture [8,21,24,26,51,52,53,54,55]. Refolding of inclusion bodies yielded soluble PanLipΔN (~0.35 mg·L^−1^); however, the full-length PanLip containing the putative propeptide region precipitated upon removal of denaturant and could not be recovered in a soluble form (Figure S3). These findings are consistent with the improved solubility of the truncated variant during heterologous expression. Similar obstacles in soluble expression of lipases containing propeptide in *E. coli* have been reported [56,57]. For example, attempts to express the pre-pro form of *Rhizopus delemar* lipase in *E. coli* resulted in inactive insoluble inclusion bodies, requiring denaturation in 8 M urea followed by redox-assisted refolding to recover active mature lipase [56]. Similarly, for *R. oryzae* lipase (ROL), successful soluble expression or refolding has often required expression in Origami (DE3) strain or secretion systems optimized for fungal lipases [57]. In the case of PanLip, the inability to recover soluble full-length protein suggests that the native propeptide may hinder folding or require host-specific maturation machinery (e.g., secretion chaperones, proteolytic processing) for correct folding, assembly, or solubility. Therefore, the role of the propeptide in PanLip’s folding, stability, or secretion supports further experimental investigation.

Far-UV CD spectra of purified PanLipΔN indicated a predominantly α/β architecture (Figure 3b). Deconvolution of the spectrum yielded 34% α-helix, 14.4% β-strand, and 52.2% other (loops/turns/disordered) [58], in close agreement with the Alphafold model-based composition (37% α-helix, 12.4% β-strand, 49.4% others). The result supports that the recombinant protein is properly folded after purification.

### 2.3. Biochemical Characterization of PanLipΔN

**Figure 4:**
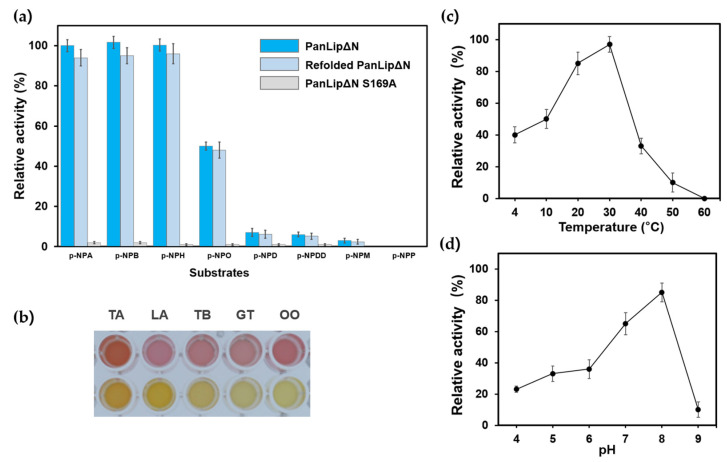
Substrate specificity and catalytic optima of PanLipΔN proteins. (**a**) Substrate specificity. Relative activity toward *p*-nitrophenyl (*p*-NP) esters of varying acyl chain length (C2-C18). Activities were normalized to the activity of *p*-NPA. (**b**) The pH indicator-based colorimetric assay to monitor the hydrolytic activity of PanLipΔN toward tertiary alcohol esters and natural substrates. TA, α-terpinyl acetate; LA, linalyl acetate; TB, glyceryl tributyrate; GT, glyceryl trioleate; OO, olive oil. Temperature (**c**) and pH (**d**) profiles. Enzyme activity measured using *p*-NPA as a substrate from 4 to 60 °C under standard assay conditions to determine the optimal temperature and from pH4-9 to identify the optimal pH.

The substrate specificity of recombinant PanLipΔN toward *p*-nitrophenyl esters with varying acyl chain lengths (C2–C16) was determined (Figure 4a). Among the tested substrates, PanLipΔN exhibited the highest catalytic activity toward *p*-nitrophenyl acetate (C2, *p*-NPA), which was set as 100% relative activity. Activity gradually decreased as the acyl chain length increased, indicating a preference for short-chain esters. Specifically, the enzyme retained activity toward *p*-nitrophenyl butyrate (C4), and *p*-nitrophenyl hexanoate (C6) but showed markedly lower activity toward medium- and long-chain substrates such as *p*-nitrophenyl octanoate (C8), *p*-nitrophenyl dodecanoate (C12), and *p*-nitrophenyl palmitate (C16) and so on. This pattern suggests that PanLipΔN functions more efficiently on short, soluble ester substrates rather than bulky hydrophobic esters. Such substrate-length preference is typical of esterase-type lipolytic enzymes, whose active sites are generally exposed to the aqueous phase and not optimized for interfacial activation [5,8,18,21,59,60,61,62]. The insoluble PanLipΔN was successfully refolded by stepwise dialysis, and the refolded enzyme exhibited activity towards *p*-NP esters that were highly comparable to those of soluble PanLipΔN (Figure 4a). This indicates that the refolding restored the catalytic function of the enzyme. To further verify the catalytic triad, we generated the PanLipΔN S169A mutant by substituting the predicted nucleophilic Ser169 with alanine (Figure 3a). This mutant exhibited no detectable activity toward any *p*-nitrophenyl ester substrate (Figure 4a), providing direct experimental evidence that Ser169 is essential for catalysis and functions as the nucleophilic component of the predicted Ser–Asp–His triad. As shown in Figure 4b, PanLipΔN also demonstrated hydrolytic activity toward bulky tertiary alcohol esters, including linalyl acetate and α-terpinyl acetate, indicating that the enzyme can accommodate sterically demanding substrates. In addition, glyceryl tributyrate (TB), glyceryl trioleate (GT), and olive oil (OO) produced a visible yellow color in the colorimetric assay, confirming that PanLipΔN is capable of hydrolyzing natural triglycerides and displays measurable lipase-like activity. This ability to act on structurally diverse and bulkier substrates may reflect an increased conformational flexibility near the active site, a feature commonly associated with cold-adapted lipolytic enzymes [63] and consistent with psychrophilic catalytic strategies that compensate for reduced thermal energy [15]. These results further support the classification of PanLipΔN as a lipase-family esterase with both esterase-type substrate preference and lipase-like catalytic capability.

The optimal temperature for hydrolytic activity of PanLipΔN was found to be at ~25 °C (Figure 4c). The effect of pH on the catalytic activity of PanLipΔN was investigated in the pH range of 4.0–9.0 (Figure 4d). The enzyme exhibited relatively low activity under acidic conditions (pH 4.0–6.0), but the activity increased sharply at near-neutral to alkaline pH. The maximum activity was observed at pH 8.0, indicating that PanLipΔN is an alkaline-preferring lipase. A rapid decline in activity was detected beyond pH 8.0, with less than 15% residual activity at pH 9.0.

**Figure 5:**
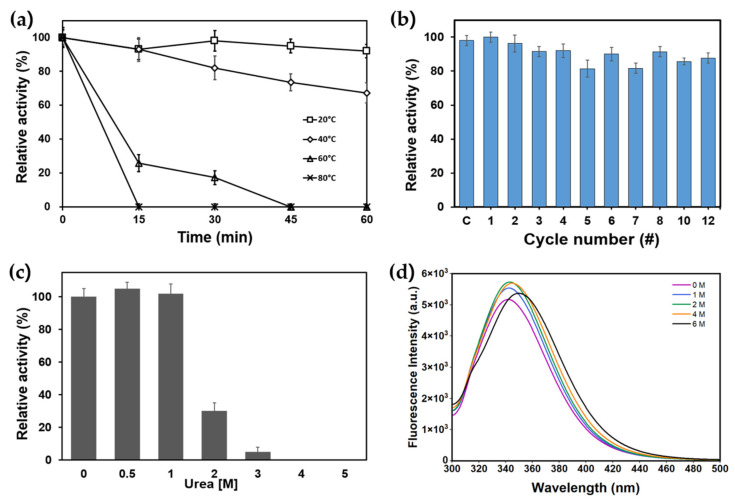
Thermal and conformational stability of PanLipΔN. (**a**) Thermal stability. Residual activity after incubation at 20, 40, 60, and 80 °C for up to 60 min, expressed as relative activity (%) normalized to time 0. (**b**) Freeze–thaw stability experiments. Enzyme activity after repeated freeze–thaw cycles (up to 20 cycles); data plotted as relative activity (%) per cycle. C indicates the storage control, in which the enzyme is stored at −20 °C for an equivalent duration without repeated freeze–thaw cycles. (**c**) Chemical denaturation. Residual activity following 30 min incubation in urea (0–5 M). (**d**) Intrinsic fluorescence during urea-induced unfolding. Tryptophan fluorescence emission spectra (excitation 280 nm) recorded for PanLipΔN in 0–6 M urea, showing progressive spectral changes associated with unfolding.

The thermal stability of PanLipΔN was examined at the different temperatures (20 °C, 40 °C, 60 °C, and 80 °C) for up to 60 min (Figure 5a). The enzyme was stable below 40 °C, maintaining over 80% of its initial activity. However, activity decreased markedly at 60 °C and was completely lost at 80 °C within 15 min. These results indicate that PanLipΔN has moderate thermal tolerance, consistent with other cold-active bacterial lipases [18,21,55,59,60,62]. The freeze–thaw stability of recombinant PanLipΔN was evaluated over twelve consecutive cycles (Figure 5b). The enzyme retained over 85% of its initial activity even after 12 cycles, showing only minor fluctuations in activity throughout the process. This result indicates that PanLipΔN not only possesses excellent structural robustness and refolding capability during repeated freezing and thawing, but also is comparable to other cold-active lipolytic enzymes. For example, the cold-active SGNH-type lipase HaSGNH1 from *Halocynthiibacter arcticus* retained most of its initial activity after nine freeze–thaw cycles [64]. Similarly, the related cold-active hormone-sensitive lipase HaHSL from the same organism was shown to withstand multiple freeze–thaw cycles with limited inactivation [21]. Such stability suggests that the enzyme’s tertiary structure is not easily disrupted by temperature-induced stresses, possibly due to the presence of stabilizing intramolecular interactions such as hydrogen bonds or hydrophobic packing [17,65]. The tolerance of PanLipΔN to repeated freeze–thaw cycles enhance its potential for industrial applications in low-temperature biocatalytic processes [4,8].

The stability of recombinant PanLipΔN was evaluated in the presence of Urea (Figure 5c,d). The enzyme displayed a pronounced sensitivity to urea, with activity gradually declining as the concentration increased. More than 80% of the activity was lost at 2 M urea, and complete inactivation occurred at concentrations above 3 M (Figure 5c). This observation suggests that urea disrupts the hydrogen bonding network and tertiary structure of PanLipΔN, resulting in a loss of catalytic function. Fluorescence emission spectra (Figure 5d) were recorded at an excitation wavelength of 280 nm to monitor tertiary structural changes in PanLipΔN in the presence of increasing urea concentrations (0–6 M). With increasing urea concentration, the fluorescence intensity gradually increased, accompanied by a slight red shift in the emission maximum from approximately 338 to 345 nm. This indicates progressive exposure of tryptophan residues as the protein structure unfolded. The shift toward longer wavelengths suggests disruption of the native tertiary structure and increased solvent accessibility [21,62,66]. Even at 6 M urea, the emission spectrum retained a discernible peak, implying that PanLipΔN still preserves partial structural integrity or folding intermediates under strongly denaturing conditions.

**Figure 6:**
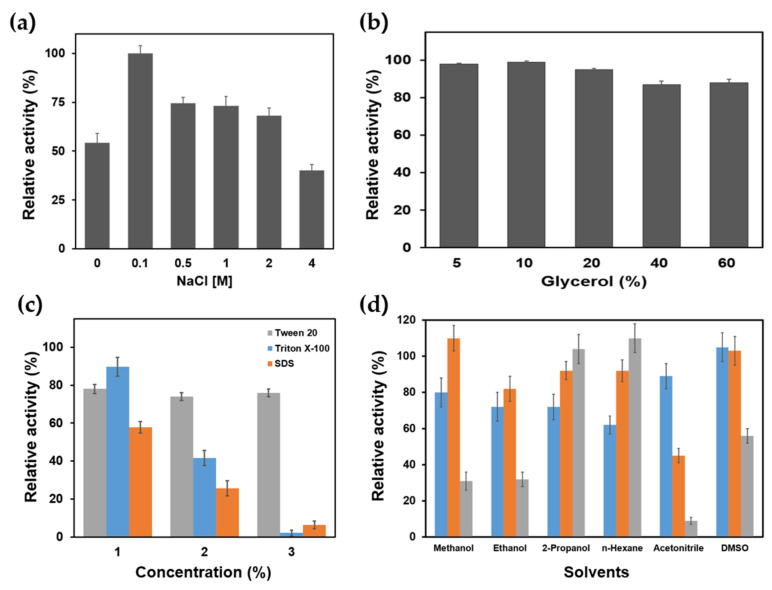
Effects of NaCl, glycerol, surfactants, and solvents on PanLipΔN activity. Enzyme activity was measured under standard assay conditions and expressed as relative activity (%) normalized to the no additive control. (**a**) NaCl dependence (0–4 M). (**b**) Glycerol dependence (5–60%, *v*/*v*). (**c**) Effect of surfactants, Tween 20, Triton X-100, and SDS, each tested at 1%, 2%, and 3% (*w*/*v* or *v*/*v* as indicated for the reagent). (**d**) Stability of PanLipΔN against various concentrations of organic solvents: 10 (blue), 25 (orange), 50% (gray) for methanol, ethanol, 2-propanol, n-hexane, and acetonitrile; 1 (blue), 2 (orange), 3 (gray) for DMSO.

The stability of recombinant PanLipΔN was evaluated in the presence of common additives such as NaCl and glycerol. PanLipΔN shows maximum activity at 0.1M NaCl, then its activity declines progressively as salt concentration increases (Figure 6a). Low concentrations slightly enhanced the activity, possibly by stabilizing the electrostatic environment or promoting proper folding, while higher NaCl concentration may disrupt the essential ion pairs or perturbs interfacial binding [18,21,25,55,60,67]. The cold-active SGNH-type lipase HaSGNH1 from *H.* showed comparable activity to PanLipΔN, retaining ~50% activity at 2.0 M NaCl [64]. In contrast, the cold-active lipase/esterase HaHSL from *H. arcticus* retains approximately 100% of its activity between 0.1 and 2.0 M NaCl, reflecting markedly higher halotolerance than PanLipΔN [21]. These comparisons indicate that PanLipΔN is less salt-tolerant than some halophilic lipases such as LipS2 from *Chromohalobacter canadensis* [68] or lipase from *Marinobacter litoralis* [69], but still functional under low to moderate salinity, which may be adequate for many aqueous or mildly saline bioprocesses. Glycerol had a mild protective effect on PanLipΔN activity (Figure 6b). The enzyme retained over 90% of its activity in 5–60% glycerol and showed a gradual decline at concentrations above 40%. This stabilizing behavior is commonly attributed to glycerol’s ability to enhance hydrophobic interactions and reduce protein flexibility, thereby maintaining the folded state under mild stress [20,21,55,67,70]. The glycerol tolerance of PanLipΔN is comparable with that of HaHSL [21], which also shows almost no loss of activity even at 60% glycerol, and higher than that of HaSGNH1, which exhibits maximal activity around 20% glycerol [64]. The slight tolerance to salt and glycerol suggests potential applicability in moderate salinity or cryoprotectant-containing biocatalytic systems [71,72].

The stability of PanLipΔN in the presence of surfactants was investigated using non-ionic (Tween 20, Triton X-100) and anionic (SDS) detergents (Figure 6c). PanLipΔN retained full activity at 0.5% Tween 20, suggesting a mild stabilizing or emulsifying effect at low concentration. However, its activity gradually decreased with increasing concentrations, maintaining approximately 80% activity at 1–3% Tween 20. This indicates that higher surfactant concentrations slightly perturb the enzyme’s hydrophobic core or substrate-binding region [73,74,75]. Triton X-100 exhibited a strong inhibitory effect in a concentration-dependent manner, with only ~85% activity remaining at 1%, ~40% at 2%, and complete inactivation at 3%. On the contrary, the cold-active HaHSL from *H. arcticus* retained >30% activity even at 5% Triton X-100, whereas it almost completely lost its activity [21]. However, HaSGNH1 lipase only ~30% of retained its activity at 0.1% Triton x-100, and less than 10% at 0.1% Tween 20 [64]. The anionic detergent SDS caused severe denaturation, reducing enzyme activity to ~55% at 1%, ~25% at 2%, and nearly zero at 3%, consistent with the disruptive nature of ionic surfactants on protein folding [76,77]. Compared to other cold-active lipases that are easily inactivated by 0.1% SDS [21,53], PanLipΔN is somewhat resistant to SDS. Notably, certain mesophilic or thermotolerant lipases such as those from *Acinetobacter* sp. [78], *Leuconostoc mesenteroides* [79], and *Bacillus* sp. RN2 [80] demonstrate substantially higher compatibility with non-ionic surfactants (Tween 20, Tween 80, Triton X-100) and SDS than is typically observed for psychrophilic lipolytic enzymes.

DMSO (1–3%) The enzyme maintained over 100% activity at 1–2% DMSO, with a moderate decline to approximately 55% at 3%. These results indicate that PanLipΔN exhibits good tolerance toward mild non-ionic surfactants and low levels of organic solvents, but is highly sensitive to strong amphiphilic or ionic detergents such as Triton X-100 and SDS.

The effect of various organic solvents on the activity of PanLipΔN was evaluated to assess its stability in solvent rich environments (Figure 6d). The effects of organic solvents, including methanol, ethanol, 2-propanol, *n*-hexane, and acetonitrile at 10, 25, and 50% (*v*/*v*) concentrations, and DMSO at 1, 2, and 3% were investigated. PanLipΔN retained substantial activity in low concentrations of short chain alcohols (Figure 6d). In 10% methanol, approximately 85% of the activity remained, indicating a transient stabilizing or substrate-diffusion effect at lower exposure levels [74,81]. However, activity sharply decreased at higher concentrations, with only residual activity observed at 50%. A similar pattern was observed for ethanol. In contrast, 2-propanol and *n*-hexane, a nonpolar solvent, showed remarkable compatibility with PanLipΔN. The enzyme retained over 70–100% activity even at 50% solvent concentration, indicating excellent tolerance to nonpolar environments and potential utility in biphasic or organic phase catalysis. Acetonitrile, however, strongly inhibited the enzyme, with less than 20% activity remaining under all conditions, likely due to its high polarity and ability to penetrate the enzyme’s interior, disrupting essential hydrogen bonding networks. In DMSO (1–3%), the enzyme maintained over 100% activity at 1–2%, with a moderate decline to approximately 55% at 3%. PanLipΔN demonstrated moderate tolerance toward low concentrations of polar alcohols and excellent stability in nonpolar solvents such as *n*-hexane, but was highly sensitive to strongly polar aprotic solvents like acetonitrile. These results suggest that PanLipΔN possesses structural rigidity favorable for catalysis in hydrophobic or low-water environments, supporting its potential application in organic phase biotransformations [4,8].

**Figure 7:**
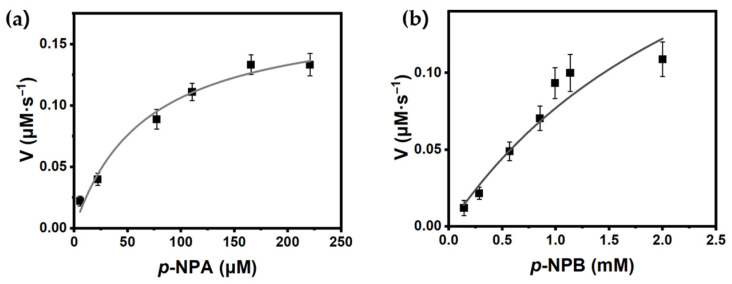
Michaelis–Menten kinetics of PanLipΔN toward *p*-nitrophenyl acetate (**a**) and butyrate (**b**). Initial velocities were plotted as function of *p*-NP esters. The kinetic parameters of PanLipΔN were determined by nonlinear least-square fits to Michaelis–Menten equation.

The kinetic parameters of PanLipΔN for *p*-NPA and *p*-NPB were determined (Figure 7). For *p*-NPA (Figure 7a), the kinetic parameters, V_max_, *K_M_*, *k_cat_*, *k_cat_*/*K_M_* were 0.18 ± 0.02 µM·s^−1^, 66.8 ± 18.5 µM, 16.2 ± 1.8 s^−1^, and 2.4 × 10^5^ M^−1^·s^−1^, respectively. The *K_M_* value suggests that *p*-NPA binds to PanLipΔN relatively tighter than many cold-active counterparts that report mM range *K_M_* for short *p*-NP esters. In contrast, *p*-NPB (Figure 7b) showed a lower *k_cat_* (9.7 ± 1.8 s^−1^) and much weaker binding (*K_M_* = 2.83 ± 1.56 mM), yielding *k_cat_*/*K_M_* 5.94 × 10^4^ M^−1^·s^−1^. These values in this study are within the range reported for cold-active microbial lipases measured on *p*-NP esters [18,21,60,62,82]. This study primarily reports kinetic parameters for short-chain model substrates, and detailed kinetic analysis using natural triglycerides remains to be completed.

### 2.4. Structural Analysis of Alphafold Generated PanLip Model

The advent of highly accurate protein structure prediction tools, particularly AlphaFold, has revolutionized structural biology by enabling detailed analyses of proteins lacking experimentally determined structures. The three-dimensional structure of PanLip was obtained from the AlphaFold Protein Structure Database (entry AF--A0A852WCG0--F1) for structural evaluation [83]. The model was generated by AlphaFold, a deep learning–based platform that produces high-accuracy protein structure predictions, and its reliability was assessed using AlphaFold’s internal confidence metric, the predicted Local Distance Difference Test (pLDDT) score. The pLDDT is a per-residue confidence value ranging from 0 to 100, which estimates the accuracy of the local structural environment of each amino acid residue.

**Figure 8:**
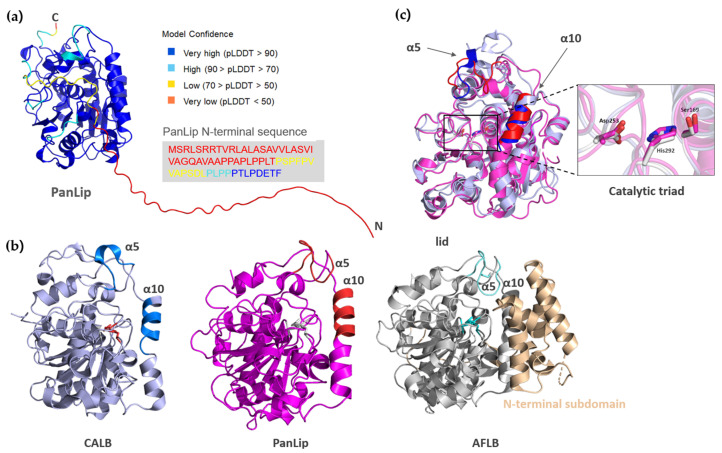
(**a**) Predicted three-dimensional structure of PanLip and residue-specific confidence estimation. The tertiary structure of PanLip was predicted using AlphaFold. The AlphaFold-generated model was displayed and colored according to its predicted local distance difference test (pLDDT) scores: blue (>90, very high confidence), cyan (70–90, confident), yellow (50–70, low confidence), and orange-red (<50, very low confidence). In this model, the N-terminal signal peptide and proline-rich propeptide regions exhibited lower prediction confidence, while the core domain, including the conserved α/β-hydrolase fold and the catalytic triad (Ser169–Asp253–His292), is predicted with very high confidence. The N-terminal residues of PanLip are also colored according to their pLDDT scores. (**b**) Ribbon presentation of structural homologs, CALB (PDB ID: 4K6H) and AFLB (PBD ID: 6IDY), and PanLip. The N-terminal residues are eliminated in this presentation of PanLip. The catalytic triad was colored in red for CALB, in gray for PanLip, and in cyan for AFLB. The regions involved in modulating substrate access in CALB (α5 and α10), PanLip (α5 and α10), and AFLB (lid) were colored in blue, red, and cyan, respectively. The N-terminal subdomain mediating AFLB activity was colored in wheat. (**c**) Superposition of PanLipΔN and CALB. PanLipΔN is in magenta; CALB in light blue. The catalytic triad is enlarged.

The full-length PanLip model (377 residues) demonstrated overall high confidence, with 78.5% of residues showing pLDDT scores above 90, indicative of a reliable structural prediction (Figure 8a). In contrast, the N-terminal signal peptide and the adjacent unique proline-rich propeptide region exhibited low to very low pLDDT value, suggesting structural disorder or intrinsic flexibility. When the N-terminal region of PanLip is eliminated, 92.5% of residues exhibited very high confidence (pLDDT > 90), particularly within the catalytic core. Further validation of the AlphaFold model was performed using Molprobity [84]. The structure achieved a clashscore of 0.91 and a MolProbity score of 1.25, ranking within the 99th percentile for models of comparable resolution. The Ramachandran plot showed 94.4% of residues in favored regions, with only 0.27% classified as outliers, confirming excellent stereochemical quality. For further structural analysis, the first 57 residues including a signal peptide and proline-rich regions were eliminated in the structural model.

Structural homolog search of the PanLip using the DALI server [85] revealed that the lipase shares the highest structural homology with several fungal lipases (Table 1), corroborating the initial sequence-based analysis. The highest-scoring homolog was lipase B from *A. fumigatus* (AFLB; PDB ID: 6IDY), with a Z-score of 56.0 and 31% sequence identity. Other significant structural homologs included lipases from *L. theobromae* (PDB ID: 7V6D; Z-score = 47.7), and *C. antarctica* (CALB; PDB ID: 4K6G; Z-score = 40.3). Notably, the highest-ranking bacterial lipase, from *Streptomyces* sp. W007 (PDB ID: 5H6B), appeared fourth in the results with a substantially lower Z-score of 24.8. These results strongly suggest that the actinomycetes PanLip adopts a fold more closely related to this family of fungal lipases than to known bacterial lipases, despite its prokaryotic origin.

The overall structure of PanLip adopts the canonical α/β-hydrolase fold characteristic of most lipases, comprising a central β-sheet core surrounded by α-helices (Figure 8b). This fold houses the catalytic triad, which in PanLip is predicted to consist of Ser169, Asp253, and His292. A key point of comparison among its structural homologs, AFLB and CALB, is the nature of a “lid” domain, a mobile element that often covers the active site and modulates substrate access [31,32]. The top structural homolog, AFLB, possesses a large, unique N-terminal subdomain and a tightly closed large lid that regulates its activity. In contrast, CALB has a short lid (Leu140-Ala146) forming α5 helix (Figure 8c). The AlphaFold model of PanLip reveals a structure that appears to be intermediate between these two lipases. It does not possess the large, complex lid of AFLB, but it does feature a flexible loop, corresponding to α5 of CALB surrounding the entrance to the active site, reminiscent of the more open architecture of CALB (Figure 8c). This structural arrangement suggests that PanLip may not rely on large-scale conformational changes for interfacial activation. However, the proline-rich N-terminal segment located adjacent to the catalytic pocket may be presumed to participate in regulating substrate accessibility. However, since the mature form of PanLip could not be expressed in a soluble state, the functional role of this proline-rich region remains to be elucidated.

### 2.5. Analysis of MD Simulation of PanLip Model

The conformational flexibility of enzymes is intrinsically linked to their catalytic activity and stability, particularly in response to temperature fluctuations. In this study, molecular dynamics (MD) simulations for 25 ns at 283, 303, 323K and CABS-flex 3.0 at different temperature parameters were performed and provided valuable insights into these dynamic properties of PanLip.

**Figure 9:**
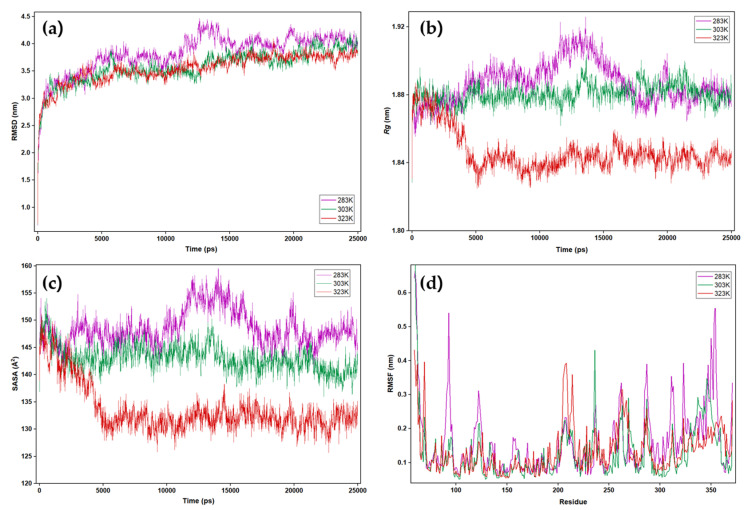
MD simulation analysis of PanLip at different temperatures: 283 K (red), 303 K (green), and 323 K (purple). (**a**) the root mean square deviation (RMSD). (**b**) the radius of gyration (Rg). (**c**) solvent-accessible surface area (SASA). (**d**) The root mean square fluctuation (RMSF) per residue.

Contrary to the common expectation that higher temperature broadens the conformational ensemble [42,59,62,94], PanLip showed its largest RMSD at 283 K (3.827 ± 0.361) and progressively smaller RMSD at 303 K (3.582 ± 0.298) and 323 K (3548 ± 0.266), indicating that the protein explores broader conformational space at low temperature (Figure 9a). The radius of gyration (Rg) is a standard compactness metric in MD; lower Rg denotes a more compact, often more rigid conformation, whereas higher Rg reflects expansion and flexibility [95,96]. The radius of gyration (Rg) also followed the same trend (Figure 9b): modestly larger Rg at lower temperatures—1.887 ± 0.012 nm (283 K) and 1.881 ± 0.006 nm (303 K)—compared with a smaller Rg at 323 K (1.846 ± 0.011 nm). Similarly, SASA traces (Figure 9c) were exhibited higher at low temperatures. The larger SASA at cold temperature reflects a slightly more solvent-exposed/relaxed surface, in line with the “surface softness” often reported for cold-adapted enzymes [17,44,97]. Together, these results support the “surface softness” model of psychrophilic proteins, in which small increases in surface breathing and solvent exposure help sustain catalytic competency at low temperature without large disruption of the catalytic core. Comparable trend has been observed for other psychrophilic enzymes and cold-active lipases, such as *G. antarctica* lipase [42], AMS8 lipase [62,98], cold-active lipase 4K6H [32].

Per-residue RMSF profiles highlighted several surface exposed loops with enhanced mobility at lower temperature, notably Thr92–Ala94, Asp122–Leu124, Rhr286–Ala288, Asp311–Asp313, and Thr346-Ala354 (Figure 9d), while the hydrophobic core remained comparatively rigid. Among them, Thr286–Ala288 lies adjacent to His292 of catalytic triad, suggesting a plausible allosteric coupling whereby increased local breathing could modulate accessibility, orientation, or proton relay efficiency at the active site, analogous to observations in other cold-active enzymes where surface softness—rather than exaggerated active-site disorder—supports catalysis at low temperature. This interpretation aligns with structural analyses of psychrophilic enzymes showing that cold adaptation frequently arises from softer surfaces with weakened inter-residue hydrogen-bond networks and restricted large-scale motions, while active-site geometries remain comparatively conserved relative to mesophilic homologs [59,99]. Residues Thr346–Ala354 of PanLip, assigned to α10, display pronounced temperature-dependent flexibility. This segment topologically matches with the residues Leu278–Ala287 of CALB α10 helix (Figure 9b), one of two short, mobile helices (α5/α10) that flank the CALB active site entrance and act as a dynamic gate for substrate access [100].

**Figure 10:**
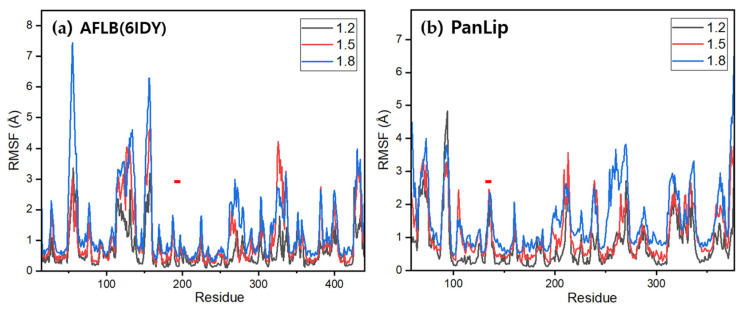
Per-residue root mean square fluctuation (RMSF) profiles of a mesophilic lipase (AFLB, PDB: 6IDY) and a psychrophilic lipase (PanLip) at three relative temperatures. Panels show RMSF (Å) per residue computed from CABS-flex ensembles for (**a**) AFLB and (**b**) PanLip. Gray, red, and blue traces correspond to relative temperatures 1.2, 1.5, and 1.8, respectively (approximating 10 °C, 30 °C, and 50 °C). The x-axis denotes residue index; the y-axis denotes RMSF (Å). In both enzymes, RMSF increases with temperature, with the largest peaks localized to solvent-exposed loops and putative lid/channel-adjacent segments, whereas secondary-structure cores remain comparatively rigid. Short horizontal red ticks highlight regions corresponding to VDLPGRS motif.

CABS-flex analyses [101] recapitulated the greater intrinsic flexibility of PanLip at lower temperature parameters and localized peaks in surface regions, while preserving the α/β-hydrolase core (Figure 10). The mesophilic AFLB exhibited a progressive increase in overall flexibility with rising temperature parameters (Figure 10a), reflected in the escalating average RMSF values: 0.68 Å at 1.2, 1.04 Å at 1.5, and 1.37 Å at 1.8. PanLip also demonstrated an increase in overall flexibility with rising temperature parameters (Figure 10b), with average RMSF values of 0.87 Å at 1.2, 1.21 Å at 1.5, and 1.73 Å at 1.8. However, its dynamic response exhibited notable differences compared to AFLB. At the lowest temperature parameter (1.2), this psychrophilic lipase displayed a high intrinsic flexibility, particularly in its N-terminus (residues 92–94, RMSF ~4.8 Å), which is significantly higher than any residues in AFLB at the same parameter. However, the region corresponding to the VDLPGRS motif (displayed as red bar on top of residues Val132-Ala137) in PanLip did not show temperature dependent fluctuations, arguing against a universal role for that this motif as a cold-adaptation determinant [42].

## 3. Materials and Methods

### 3.1. Chemicals and Reagents

The polymerase chain reaction (PCR) premix kit, plasmid purification kit, restriction enzymes, and T4 DNA ligase was purchased from Bioneer (Daejeon, Republic of Korea). PCR cleanup and gel extraction kit were obtained from Takara Biomedicals (Seoul, Republic of Korea). Synthesis of primers and DNA sequencing were performed by Macrogen (Seoul, Republic of Korea). Linalyl acetate, α-terpinyl acetate, tert-butyl acetate, glyceryl trioleate, olive oil, *p*-nitrophenyl acetate, *p*-nitrophenyl butyrate, *p*-nitrophenyl hexanoate, *p*-nitrophenyl octanoate, *p*-nitrophenyl decanoate, *p*-nitrophenyl dodecanoate, *p*-nitrophenyl myristate, and *p*-nitrophenyl palmitate were obtained from Sigma-Aldrich (St. Louis, MO, USA). The Ni^2+^ affinity resin was purchased from Qiagen (Hilden, Germany). The 96-well PCR plate (Code 781368) was obtained from Axygen (Corning, NY, USA). All other chemicals and reagents used in this study were of analytical grade unless otherwise stated.

### 3.2. Cloning, Expression, and Purification of Pseudonocardia Antarctica Lipolytic Enzyme

The signal peptide-deleted lipase gene for *Pseudonocardia antarctica* lipolytic enzyme, designated PanLip, was codon-optimized and synthesized by GenScript (Piscataway, NJ, USA). The gene was cloned into three different expression vectors, pET-22b(+), pET-28a(+), and pET-32a(+) vectors, to compare the expression levels of recombinant proteins. To improve the solubility of PanLip, which was largely expressed as inclusion bodies in both *E. coli* BL21(DE3) and SHuffle strains, an N-terminally truncated mutant, designated PanLipΔN, was designed by removing the first 27 amino acid residues of the full-length enzyme. PanLipΔN was obtained by amplifying the truncated fragment from the wild-type PanLip gene. To construct PanLipΔN1, the gene was amplified using the following primers in Table 2. PCR amplification was performed in a 20 μL reaction mixture containing 1× PCR premix (Bioneer, Daejeon, Republic of Korea), 0.2 μM of each primer, and the template DNA. The cycling conditions were as follows: initial denaturation at 95 °C for 3 min; 30 cycles of 95 °C for 30 s, 58 °C for 30 s, and 72 °C for 60 s; and a final extension at 72 °C for 7 min. The amplified product was verified by 1% agarose gel electrophoresis and purified using the DNA fragment purification kit (Takara). The purified Δ27N PCR product and pET-28a(+) vector were digested with NdeI and XhoI, gel-purified, and ligated using T4 DNA ligase (Bioneer, Daejeon, Republic of Korea) at a 3:1 insert-to-vector molar ratio. The ligation product was transformed into *E. coli* DH5α competent cells for plasmid propagation. Positive clones were confirmed by colony PCR and restriction digestion analysis. The insert sequence was verified by Sanger sequencing (Macrogen, Seoul, Republic of Korea). Site-directed mutagenesis of Ser 169 to Ala was conducted using the QuikChange site-directed mutagenesis kit (Stratagene, LaJolla, CA, USA) according to the manufacturer’s instructions.

To evaluate the solubility improvement of truncated variants, the confirmed recombinant plasmids PanLipΔN were transformed into *E. coli* SHuffle T7 Express cells for expression. The transformants were selected on LB agar plates containing 50 μg·mL^−1^ kanamycin. A single colony was inoculated into 5 mL of LB medium and cultured overnight at 37 °C with shaking at 190 rpm. The seed culture was transferred to 2L of fresh LB medium and incubated until OD_600_ reached 0.6–0.8. Protein expression was induced by adding 0.1 mM IPTG and incubating at 20 °C for 16 h.

Cells were harvested by centrifugation (12,000 rpm, 15 min, 4 °C), resuspended in lysis buffer (50 mM sodium phosphate, pH 8.0, 400 mM NaCl, 10 mM imidazole), and disrupted by sonication on ice for 5 min with 5 s pulses followed by 5 s intervals. After centrifugation (12,000 rpm, 30 min, 4 °C), the soluble and insoluble fractions were analyzed by SDS–PAGE. The ΔN variants showed markedly improved solubility compared with the full-length PanLip, particularly in the SHuffle strain.

For purification, the soluble fraction was loaded onto a Ni^2+^–NTA affinity column (Qiagen, Germany) pre-equilibrated with binding buffer (50 mM sodium phosphate, pH 8.0, 300 mM NaCl, 10 mM imidazole). After washing the column with 40 mM imidazole buffer to remove nonspecifically bound proteins, the target protein was eluted stepwise with imidazole concentrations of 100 mM, 150 mM, 200 mM, and 250 mM in the same buffer. Eluted fractions were analyzed by SDS–PAGE, desalted, and concentrated and buffer-exchanged using Amicon Ultra filters (10 kDa cutoff). The purified concentration is determined by the Bradford assay [102]. The insoluble fractions of PanLip and PanLipΔN were subjected to refolding to recover soluble, active enzyme using a stepwise dialysis refolding method as described previously with slight modifications [103]. After cell lysis, the inclusion bodies were collected by centrifugation and washed twice with washing buffer (20 mM Tris–HCl, pH 8.0, 0.5% Triton X-100, 1 mM EDTA), followed by a final wash with 20 mM Tris–HCl (pH 8.0). The washed pellets were solubilized in 8 M urea (50 mM Tris–HCl, pH 8.0, 300 mM NaCl) and purified under denaturing conditions using a Ni^2+^–NTA affinity column. The eluted fraction was reduced by adding 50-fold molar access of β-mercaptoethanol (β-ME). β-ME was removed by dialysis against 8 M urea (50 mM Tris–HCl, pH 8.0, 300 mM NaCl) without β-ME. The unfolded PanLip proteins (~2 mg/mL in 8 M urea) were transferred into dialysis tubing, and refolding was carried out at room temperature (22–25 °C) by stepwise dialysis against refolding buffer (50 mM Tris–HCl, pH 8.0, 1 mM EDTA, 1% Triton X-100, and 0.3 M L-arginine-HCl) while gradually decreasing the urea concentration (4 M, 2 M, 1 M, 0.5 M, and 0 M). At the 2 M urea step, a redox couple (2 mM reduced glutathione and 0.2 mM oxidized glutathione) was added to facilitate disulfide bond formation. The sample was maintained overnight at 1 M urea and subsequently dialyzed sequentially to 0.5 M and 0 M urea. Following complete removal of urea, the solution was clarified by centrifugation (20,000× *g*, 15 min) and filtered through a 0.22 µm membrane. The refolded protein was concentrated using an Amicon Ultra centrifugal filter unit (10 kDa cutoff).

### 3.3. Enzyme Activity Assay of Recombinant PanLipΔN

#### 3.3.1. Substrate Specificity

The substrate specificity of recombinant PanLipΔN was determined using *p*-nitrophenyl esters (*p*-NP esters) with acyl chain lengths from C2 to C18. The standard reaction mixture (1.5 mL) contained 200 mM Tris–HCl pH 7.4, 100 mM NaCl, and 1 mM *p*-NP ester (dissolved in isopropanol). The reaction was started by adding the enzyme and incubated at 25 °C for 20 min. The release of *p*-nitrophenol was monitored at 405 nm and relative activity was calculated using *p*-nitrophenyl acetate (*p*-NPA) as the reference (100%). Error bars represent the standard deviation from triplicate measurements.

#### 3.3.2. Effect of pH and Temperature

The effect of pH on enzyme activity was evaluated in 200 mM Tris–HCl buffer containing 100 mM NaCl, with pH adjusted from 3.0 to 10.0 using small amounts of HCl or NaOH. Reactions were performed at 25 °C for 20 min using *p*-NPA as the substrate, and absorbance was measured at 405 nm. The effect of temperature was assessed by performing reactions from 10 °C to 70 °C in the same buffer at pH 7.4, with the activity at 25 °C taken as 100%. Thermal stability was evaluated by preincubating the enzyme at various temperatures for 30 min and determining the residual activity.

#### 3.3.3. Freeze–Thaw Stability

The enzyme was subjected to 1, 2, 4, 6, 8, 10, and 12 freeze–thaw cycles between −20 °C and 25 °C. After each cycle, residual activity was measured using *p*-NPA as the substrate under standard assay conditions. The activity before freezing was defined as 100%. Error bars represent standard deviations from three independent experiments.

#### 3.3.4. Effects of Additives

To evaluate the effect of chemical additives on PanLipΔN, enzyme samples were incubated for 30 min at room temperature in 200 mM Tris–HCl pH 7.4, 100 mM NaCl supplemented with varying concentrations of additives. Urea (0.1–5 M), NaCl (0.1–4 M), glycerol (0–60%), and Tween-20 (0.5–3%). Following incubation, residual activity was assayed using *p*-NPA at 25 °C. The activity without any additive was defined as the control (100%).

#### 3.3.5. Effects of Surfactants and Organic Solvents

The tolerance of PanLipΔN to surfactants and organic solvents was examined by preincubating the enzyme at 25 °C for 20, 40, or 60 min. The following surfactants and organic solvents are examined: Tween 20, Triton X-100, and SDS at 1–3% (*v*/*v*); methanol, ethanol, *n*-butanol, isopropanol, acetonitrile, *n*-hexane at 10–50% (*v*/*v*) and DMSO at 1–3% (*v*/*v*). After treatment, enzyme activities were determined under the standard condition, and relative activity was expressed as a percentage of the untreated control.

#### 3.3.6. Spectroscopy of PanLipΔN

A 100 μg·mL^−1^ PanLipΔN was prepared in 10 mM sodium phosphate pH 7.4, 100 mM NaCl containing the indicated urea concentrations (0, 1, 2, 4, and 6 M). Samples were equilibrated at 25 °C for 20 min prior to measurement. Intrinsic fluorescence spectra were recorded with λex of 280 nm and scanned from 300 to 450 nm in a 1 cm quartz cuvette. Buffer baselines were subtracted. The emission maximum (λmax) and peak intensity were used to evaluate tertiary structural changes as a function of urea.

Far-UV CD spectra were recorded on a Jasco J1500 circular dichroism spectrometer (Tokyo, Japan) to assess secondary structure content of PanLipΔN. A 200 μg·mL^−1^ sample in 10 mM sodium phosphate buffer pH 7.4 was placed in a 0.1 cm path-length quartz cuvette and scanned over 190–250 nm at 25 °C. Spectra were collected as an average of five scans. The secondary structure was estimated using Bestsel webserver (https://bestsel.elte.hu/ssfrompdb.php, accessed on 15 July 2025).

#### 3.3.7. Kinetic Parameter Determination

Kinetic parameters were determined using *p*-nitrophenyl acetate (*p*-NPA) and *p*-nitrophenyl butyrate (*p*-NPB) as substrate. Reactions (1 mL total volume) were carried out at 25 °C in 100 mM Tris–HCl pH 7.4, 100 mM NaCl. Initial rates were obtained from the linear region, and kinetic parameters were calculated by nonlinear regression using Origin 2024. All kinetic experiments were performed in triplicate.

### 3.4. Bioinformatic and Structural Model Analysis and MD Simulation

Physicochemical parameters of PanLip were computed by ProtParam [104]. Signal peptide was predicted using SignalP 6.0 (https://services.healthtech.dtu.dk/services/SignalP-6.0/, accessed on 3 October 2024) [105]. The amino acid sequence of *P. antarctica* lipase (PanLip; UniProt ID: A0A852WCG0) was analyzed to determine its phylogenetic position within bacterial lipolytic enzyme families. The classification framework followed that of Hitch and Clavel (2019) [46], who organized bacterial lipolytic enzymes into 35 families based on sequence similarity and function. Representative sequences from each family were retrieved from the UniProt database, and PanLip was included as the query sequence. Multiple sequence alignment was performed using the ClustalW program and phylogenetic reconstruction was conducted in MEGA 12.0.11 [106] using the Maximum Likelihood (ML) method and the Jones–Taylor–Thornton (1992) model of amino acid substitution.

The three-dimensional structure of PanLip was retrieved from the AlphaFold Protein Structure Database (entry AF--A0A852WCG0--F1). The predicted model was downloaded in PDB format. Model confidence at the local residue--level was assessed via the per--residue predicted local distance difference test (pLDDT) scores, and global structural consistency was evaluated using the predicted aligned error (PAE) matrix provided. Regions of high, moderate and low prediction confidence were annotated and color--coded according to standard ranges (e.g., pLDDT > 90, 70–90, <70). The model quality was assess using MolProbity (v4.5.2) [84]. To identify structural homologs of the PanLip, a structural similarity search was conducted against the Protein Data Bank (PDB) using the DALI server. All structural figures were prepared using PyMOL version 2.5.8.

To investigate temperature-dependent conformational flexibility of lipases, we performed CABS-flex 3.0 https://lcbio.pl/cabsflex3/; accessed on 5 June 2025) simulations [101] on mesophilic *A, fumigatus* lipase (PDB: 6IDY) and AlphaFold structure model of PanLip. Simulations were conducted with default options at relative temperatures of 1.2, 1.5, and 1.8 to approximate 10 °C, 30 °C, and 50 °C, respectively. We also performed MD simulation to study molecular dynamics of PanLip at different temperatures with GROMACS using the Neurosnap platform (https://neurosnap.ai/; accessed on 5 June 2025). AlphaFold generated PanLip structure was energy minimized and subjected to MD simulation for 25 ns at three temperatures (10 °C, 30 °C, and 50 °C). OPLS-AA/L force filed was used for all MD simulations [107].

## 4. Conclusions

This study reports identification and characterization of PanLip, a cold-active lipolytic enzyme from *Pseudonocardia antarctica*. Limited soluble expression was achieved by N-terminal truncation (PanLipΔN), enabling purification and functional analyses. Insoluble PanLipΔN was also successfully refolded. PanLipΔN displays a clear preference for short-chain *p*-nitrophenyl esters, an alkaline pH optimum, and maximal activity near 25 °C, while retaining measurable hydrolytic activity toward natural triglycerides, consistent with its classification as a lipolytic enzyme with esterase-type substrate preference. Its activity profile shows a distinct ionic-strength optimum (0.1 M NaCl) and notable compatibility with certain organic solvents, features that are advantageous for low-temperature and biphasic processes. AlphaFold and DALI analysis placed PanLip closest to fungal lipases (CALB/AFLB), and temperature series MD and CABS-flex revealed temperature-sensitive flexibility concentrated in surface loops and a region homologous to the CALB α10 helix—elements known to regulate substrate ingress/egress without large lid motions. Structure-based multiple sequence alignments did not support the VDLPGRS motif as a unique motif of cold adaptation, emphasizing that distributed flexibility and dynamic context, rather than short linear motifs, underpin low-temperature performance. Together these findings expand the emerging repertoire of the cold-active lipolytic enzymes from Antarctic actinomycetes and establish PanLip as a tractable, CALB-like scaffold for future engineering. Ongoing studies will include interfacial kinetics on emulsified substrates, and evaluation of application-relevant reactions in low-temperature and low-water media.

## Figures and Tables

**Table 1:** DALI search results.

PDB ID	Z-Score	rmsd	Identity (%)	aln/nres ^1^	Species	Reference
6IDY	56.0	0.6	31	311/421	*Aspergillus fumigatus*	[31]
7V6D	47.7	1.4	28	307/366	*Lasiodiplodia theobromae*	[86]
4K6G	40.3	1.8	30	289/319	*Candida antarctica*	[32]
5H6B	24.8	2.2	27	220/252	*Streptomyces* sp. *W007*	[87]
7V3K	24.0	2.4	25	224/294	*Janibacter* sp. *HTCC2649*	[88]
6WPY	19.3	2.7	17	201/245	*Bacillus licheniformis*	[89]
1PJA	17.9	2.5	15	193/268	*Homo sapiens*	[90]
1ISP	17.6	1.8	23	163/179	*Bacillus subtilis*	[91]
4BRS	17.5	3.2	13	220/332	*Paucimonas lemoignei*	[92]
8PI1	17.3	2.8	13	199/271	*Pseudomonas fluorescens*	[93]

^1^ aln/nres: number of residues aligned/total number of residues.

**Table 2:** Plasmids and primers used in this study.

Plasmids/Primers	Description
Plasmids	
pET-22a-PanLip	Amp ^r^; codon-optimized *P. antarctica* lipase gene inserted into NcoI/HindIII
pET-28a-PanLip	Kan ^r^; codon-optimized *P. antarctica* lipase gene inserted into NdeI/BamHI
pET-32a-PanLip	Amp ^r^; codon-optimized *P. antarctica* lipase gene inserted into NcoI/HindIII
pET-28a-PanLipΔN	Kan ^r^; N-terminal deleted codon-optimized *P. antarctica* lipase gene inserted into NdeI/XhoI
Primers	
ΔN-F	5′–GCGGCATATGACTCTGCCGGATGAGAC–3′ (underlined was NdeI)
ΔN-R	5′–GCGGCTCGAGTTATTCCAGTAGGTTCTGTTTC–3′ (underlined was XhoI)

## Data Availability

The majority of the data generated and analyzed during this study are included in this article and its supplementary materials. Additional data are available from the corresponding author upon request.

## Supplementary Materials

The following supporting information can be downloaded at: https://www.mdpi.com/article/10.3390/md23120480/s1. Figure S1: Comparative Sequence Analysis and Codon Optimization of *Pseudonocardia antarctica* Lipase and Its Propeptide Variants; Figure S2: Multiple sequence alignment (MSA) of lipases from thermophilic, mesophilic, and psychrophilic actinomycetes performed using T-Coffee Expresso based on 3D structural information; Figure S3: SDS-PAGE analysis of refolded PanLipΔN; Table S1: Similarity of Pseudonocardia antarctica lipase against the accepted type proteins for each lipolytic family.
