# Supplementary material for: A CALB-like Cold-Active Lipolytic Enzyme from Pseudonocardia antarctica: Expression, Biochemical Characterization, and AlphaFold-Guided Dynamics
Source: Mar Drugs. 2025 Dec 15;23(12):480. doi: 10.3390/md23120480 (PMC12734981; doi:10.3390/md23120480)
Supplement: Supplementary file 1 [file marinedrugs-23-00480-s001.zip › supplementary_materials_PanLipase.pdf]

Supplementary materials

A CALB-like Cold-Active Lipolytic Enzyme from *Pseudonocardia antarctica*: Expression, Biochemical Characterization, and AlphaFold-Guided Dynamics

Lixiao Liu [1], Hackwon Do [2], Jong-Oh Kim [3], Jun Hyuck Lee [2] and Hak Jun Kim [1,*]

[1] Department of Chemistry, Pukyong National University, Busan 48513, Republic of Korea; liulixiao@pukyong.ac.kr
[2] Division of Life Sciences, Korea Polar Research Institute, Incheon 21990, Republic of Korea; hackwondo@kopri.re.kr (H.D.); junhyucklee@kopri.re.kr (J.H.L.)
[3] Department of Microbiology, Pukyong National University, Busan 48513, Republic of Korea; jokim@pknu.ac.kr
* Correspondence: kimhj@pknu.ac.kr; Tel.: +82-51-629-5587

(**a**)

>tr|A0A852WCG0|A0A852WCG0_PSEA5 Lipase OS=Pseudonocardia alni OX=33907 GN=HDA37_005286 PE=4 SV=1

MSRLSRRTVRLALASAVVLASVIVAGQAVA**APPAPLPPLTPSPFPVVAPSDLPLPPPTLPDETFGPVDRP**GPALSV
PPDQLDAAVRCSANATDADRDVALFVPGTTLTPEVNFGFNWFAALDDLGRPYCSVTLPNNAMTDTQIAAEYVVHAI
RHVHGISGRKVDVLGHSQGGTEPRFALRFWPDLRGMVDDYVAFGTTNHGSIAINAALCTPATGCAEALWQQTLNSH
YTQAMNSYQETFAGISYTQIYTRTDEFVQPNLDDSGTTSLHGGGGEISNVALQDVCLTEAASEHIAVGTYSPVAYA
LATDALDHDGPADPARVDRGVCTQVFMPGVDPLTFPTDYAATLGLIANQLALAPRVGSEPELRPYTLADDRQG

(**b**)

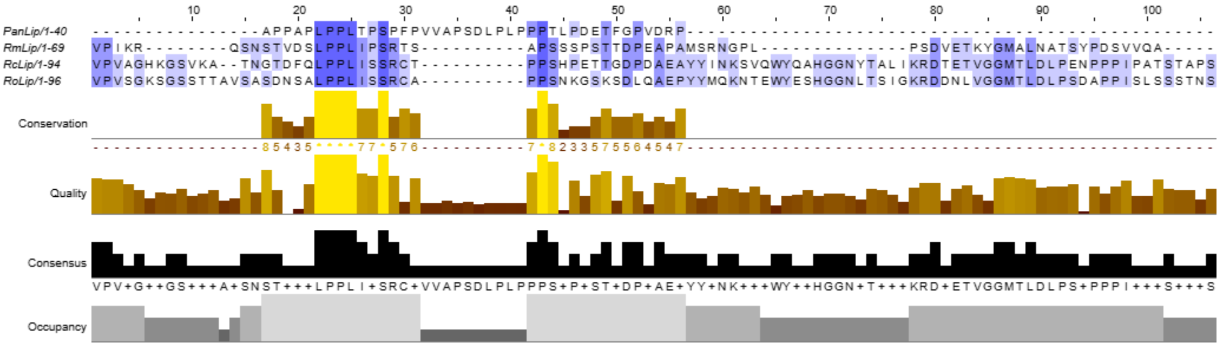

(**c**)

cat	atg	gca	ccc	ccc	gct	cca	tta	ccg	cct	cta	acg	ccg	tcg	ccg	ttt	ccg	gtt	gtg	gcg
H	M	**A**	**P**	**P**	**A**	**P**	**L**	**P**	**P**	**L**	**T**	**P**	**S**	**P**	**F**	**P**	**V**	**V**	**A**
cca	agc	gat	ctg	ccg	ctg	cct	ccg	ccg	act	ctg	ccg	gat	gag	acc	ttc	ggc	cca	gtg	gac
**P**	**S**	**D**	**L**	**P**	**L**	**P**	**P**	**P**	T	L	P	D	E	T	F	G	P	V	D
cgt	ccg	ggt	ccg	gca	ctg	agc	gta	ccg	ccg	gac	cag	ctt	gat	gcc	gct	gtg	cgt	tgt	tcc
R	P	G	P	A	L	S	V	P	P	D	Q	L	D	A	A	V	R	C	S
gcg	aac	gct	acg	gat	gcg	gat	cgt	gat	gtt	gcg	ttg	ttc	gtg	ccg	ggt	acg	acc	ttg	acc
A	N	A	T	D	A	D	R	D	V	A	L	F	V	P	G	T	T	L	T
cct	gag	gtc	aat	ttt	ggc	ttt	aac	tgg	ttc	gca	gca	ctg	gat	gac	ctg	ggc	cgc	cca	tac
P	E	V	N	F	G	F	N	W	F	A	A	L	D	D	L	G	R	P	Y
tgc	agc	gtt	acc	ttg	ccg	aac	aac	gcg	atg	acc	gat	acc	caa	atc	gcg	gca	gaa	tat	gta
C	S	V	T	L	P	N	N	A	M	T	D	T	Q	I	A	A	E	Y	V
gtt	cac	gca	att	cgt	cac	gtg	cac	ggc	atc	tct	ggc	cgt	aaa	gtg	gac	gtt	ctg	ggt	cat
V	H	A	I	R	H	V	H	G	I	S	G	R	K	V	D	V	L	G	H
agc	cag	ggt	ggt	act	gaa	ccg	cgt	ttc	gcg	ctg	cgc	ttc	tgg	ccg	gat	ctc	cgc	ggt	atg
S	Q	G	G	T	E	P	R	F	A	L	R	F	W	P	D	L	R	G	M
gtt	gac	gat	tac	gtc	gcg	ttt	ggt	acc	aca	aat	cac	ggt	tca	att	gcg	atc	aac	gca	gcg
V	D	D	Y	V	A	F	G	T	T	N	H	G	S	I	A	I	N	A	A
ctt	tgc	acc	cca	gcg	acc	ggt	tgt	gca	gaa	gcg	tta	tgg	cag	cag	acc	ctg	aac	agc	cat
L	C	T	P	A	T	G	C	A	E	A	L	W	Q	Q	T	L	N	S	H
tat	acc	caa	gcg	atg	aat	tcc	tat	caa	gaa	acc	ttc	gct	ggc	atc	tcc	tac	acc	caa	atc
Y	T	Q	A	M	N	S	Y	Q	E	T	F	A	G	I	S	Y	T	Q	I
tat	aca	cgt	acc	gac	gaa	ttt	gtg	cag	ccg	aac	ctg	gac	gac	agc	ggc	act	acg	agc	ctg
Y	T	R	T	D	E	F	V	Q	P	N	L	D	D	S	G	T	T	S	L
cat	ggt	ggc	ggt	ggc	gaa	atc	agt	aat	gtt	gcc	ctg	caa	gat	gtc	tgc	ctg	acc	gag	gcc
H	G	G	G	G	E	I	S	N	V	A	L	Q	D	V	C	L	T	E	A
gcc	agc	gag	cac	att	gcg	gtc	gga	acc	tac	agc	ccg	gtg	gcg	tat	gca	ttg	gcg	acg	gac
A	S	E	H	I	A	V	G	T	Y	S	P	V	A	Y	A	L	A	T	D
gct	ctc	gat	cat	gac	ggt	ccg	gcc	gat	ccg	gcg	cgt	gtg	gac	aga	ggg	gtg	tgc	acc	cag
A	L	D	H	D	G	P	A	D	P	A	R	V	D	R	G	V	C	T	Q
gtt	ttt	atg	ccg	ggt	gtt	gac	ccg	ctg	acc	ttc	ccg	acg	gac	tac	gcc	gct	act	ttg	ggc
V	F	M	P	G	V	D	P	L	T	F	P	T	D	Y	A	A	T	L	G
ctg	att	gcg	aac	caa	ctg	gcg	tta	gct	ccg	cgc	gtg	ggt	tct	gag	ccg	gag	ctg	cgt	ccg
L	I	A	N	Q	L	A	L	A	P	R	V	G	S	E	P	E	L	R	P
tac	acc	ttg	gct	gac	gac	cgc	cag	ggc	taa										
Y	T	L	A	D	D	R	Q	G	-										

(d)

```
act ctg ccg gat gag acc ttc ggc cca gtg gac cgt ccg ggt ccg gca ctg agc gta ccg
 T   L   P   D   E   T   F   G   P   V   D   R   P   G   P   A   L   S   V   P
ccg gac cag ctt gat gcc gct gtg cgt tgt tcc gcg aac gct acg gat gcg gat cgt gat
 P   D   Q   L   D   A   A   V   R   C   S   A   N   A   T   D   A   D   R   D
gtt gcg ttg ttc gtg ccg ggt acg acc ttg acc cct gag gtc aat ttt ggc ttt aac tgg
 V   A   L   F   V   P   G   T   T   L   T   P   E   V   N   F   G   F   N   W
ttc gca gca ctg gat gac ctg ggc cgc cca tac tgc agc gtt acc ttg ccg aac aac gcg
 F   A   A   L   D   D   L   G   R   P   Y   C   S   V   T   L   P   N   N   A
atg acc gat acc caa atc gcg gca gaa tat gta gtt cac gca att cgt cac gtg cac ggc
 M   T   D   T   Q   I   A   A   E   Y   V   V   H   A   I   R   H   V   H   G
atc tct ggc cgt aaa gtg gac gtt ctg ggt cat agc cag ggt ggt act gaa ccg cgt ttc
 I   S   G   R   K   V   D   V   L   G   H   S   Q   G   G   T   E   P   R   F
gcg ctg cgc ttc tgg ccg gat ctc cgc ggt atg gtt gac gat tac gtc gcg ttt ggt acc
 A   L   R   F   W   P   D   L   R   G   M   V   D   D   Y   V   A   F   G   T
aca aat cac ggt tca att gcg atc aac gca gcg ctt tgc acc cca gcg acc ggt tgt gca
 T   N   H   G   S   I   A   I   N   A   A   L   C   T   P   A   T   G   C   A
gaa gcg tta tgg cag cag acc ctg aac agc cat tat acc caa gcg atg aat tcc tat caa
 E   A   L   W   Q   Q   T   L   N   S   H   Y   T   Q   A   M   N   S   Y   Q
gaa acc ttc gct ggc atc tcc tac acc caa atc tat aca cgt acc gac gaa ttt gtg cag
 E   T   F   A   G   I   S   Y   T   Q   I   Y   T   R   T   D   E   F   V   Q
ccg aac ctg gac gac agc ggc act acg agc ctg cat ggt ggc ggt ggc gaa atc agt aat
 P   N   L   D   D   S   G   T   T   S   L   H   G   G   G   G   E   I   S   N
gtt gcc ctg caa gat gtc tgc ctg acc gag gcc gcc agc gag cac att gcg gtc gga acc
 V   A   L   Q   D   V   C   L   T   E   A   A   S   E   H   I   A   V   G   T
tac agc ccg gtg gcg tat gca ttg gcg acg gac gct ctc gat cat gac ggt ccg gcc gat
 Y   S   P   V   A   Y   A   L   A   T   D   A   L   D   H   D   G   P   A   D
ccg gcg cgt gtg gac aga ggg gtg tgc acc cag gtt ttt atg ccg ggt gtt gac ccg ctg
 P   A   R   V   D   R   G   V   C   T   Q   V   F   M   P   G   V   D   P   L
acc ttc ccg acg gac tac gcc gct act ttg ggc ctg att gcg aac caa ctg gcg tta gct
 T   F   P   T   D   Y   A   A   T   L   G   L   I   A   N   Q   L   A   L   A
ccg cgc gtg ggt tct gag ccg gag ctg cgt ccg tac acc ttg gct gac gac cgc cag ggc
 P   R   V   G   S   E   P   E   L   R   P   Y   T   L   A   D   D   R   Q   G
taa
 -
```

Figure S1. Comparative Sequence Analysis and Codon Optimization of Pseudonocardia antarctica Lipase and Its Propeptide Variants. The DNA sequence of (a) amino acid sequence of PanLip, (b) multiple sequence alignment of propeptide region of lipasese. PanLip: *Pseudonocardia antarctica* lipase (accession no.: WP_179762565.1); RmLip: *Rhizomucor miehei* Lipase (accession no.: CAA00250.1); RcLip: *Rhizopus chinensis* Lipase (accession no.: ABN59381.2); RoLip: *Rhizopus oryzae* Lipase (accession no.: AAF32408.1), (c) a codon-optimzed propeptide-containing *P. antarctica* lipase gene, and (d) a codon-optimzed N-terminal 27 residue deleted PanLip gene. In panel (a), the signal peptide was underlined, and the putative propeptide regions was highlighted in bold red. In panel (c), the N-terminal 27 residues subjected to deletion were highlighted in red.

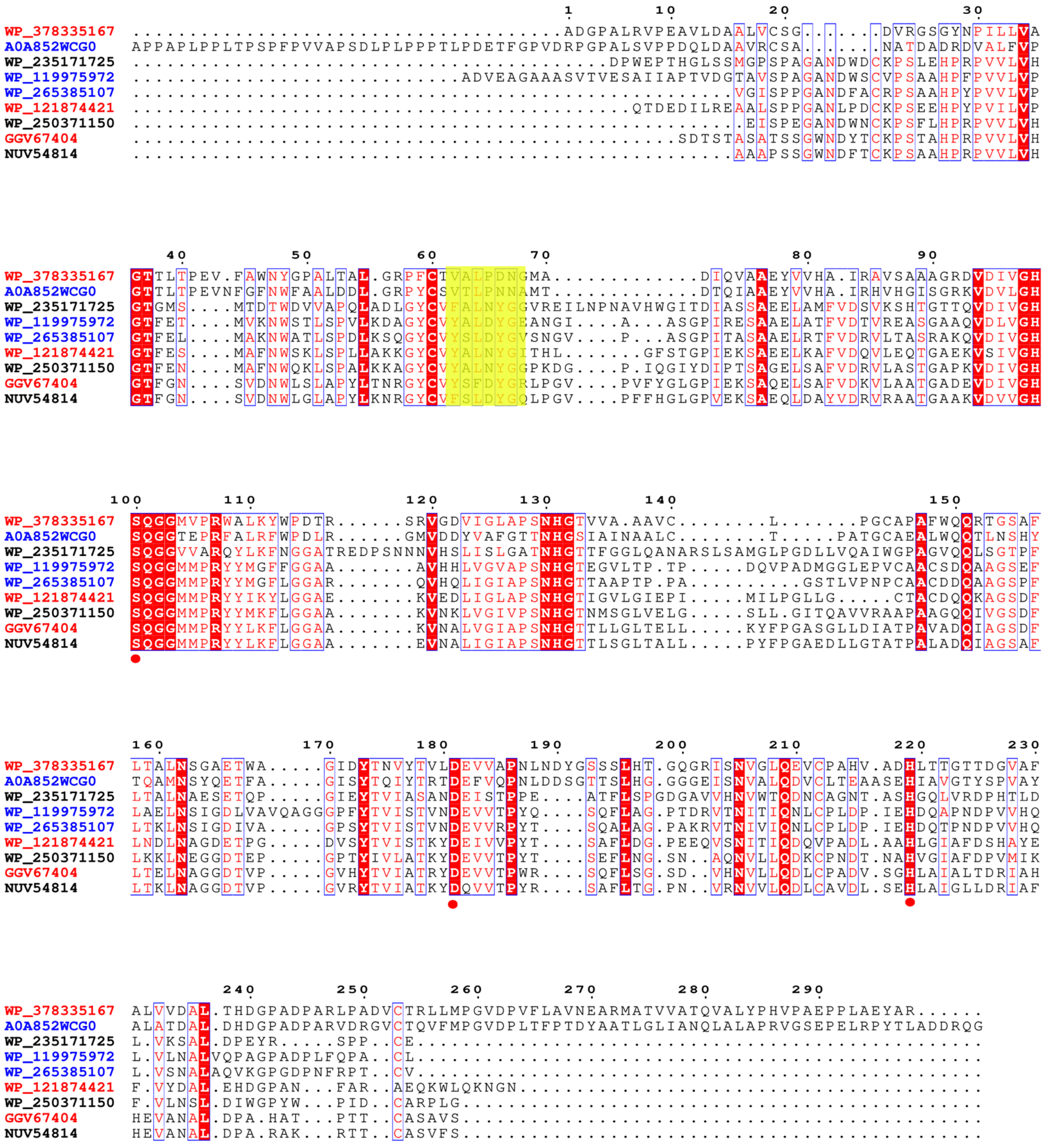

Figure S2. Multiple sequence alignment (MSA) of lipases from thermophilic, mesophilic, and psychrophilic actinomycetes performed using T-Coffee Expresso based on 3D structural information. The alignment includes three thermophilic actinomycete lipases from *Amycolatopsis thermoflava* (WP_378335167), *Thermoactinomyces vulgaris* (WP_121874421), and *Streptomyces thermoviolaceus* subsp. *apingens* (GGV67404); three mesophilic actinomycete lipases from and *Actinomadura madurae* (WP_250371150), *Rhodococcus erythropolis* (WP_235171725), and *Streptomyces coelicolor* (NUV54814); and three psychrophilic actinomycete lipases from *Cryobacterium melibiosiphilum* (WP_119975972), *Pseudonocardia antarctica* (A0A852WCG0), and *Rhodococcus antarcticus* (WP_265385107). The accession numbers for thermophilic lipases were written in red, for mesophilic in black, and for psychrophilic in blue. The motif proposed to be involved in cold adaptation are highlighted in bright yellow. The figure was generated using ESPript 3.0 (https://espript.ibcp.fr/ESPript/ESPript/: accessed on 10 March 2025).

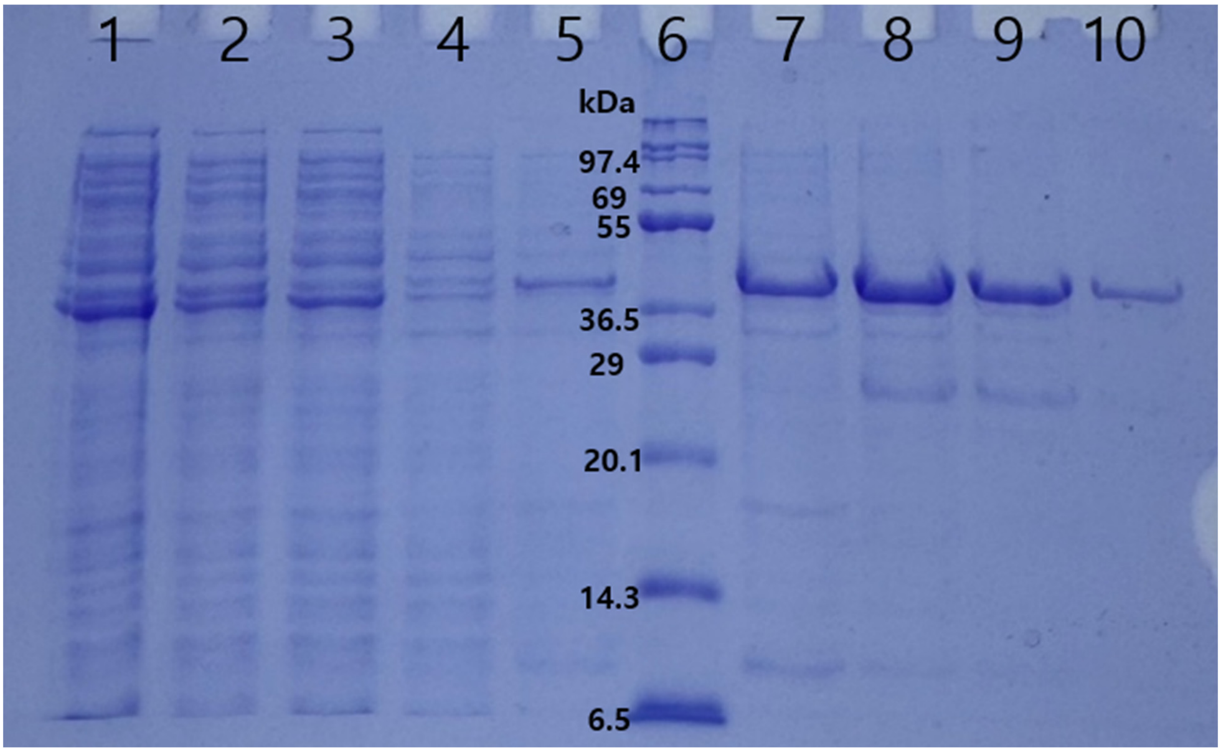

Figure S3. SDS-PAGE analysis of refolded PanLipΔN. PanLipΔN was purified from inclusion bodies and refolded by stepwise dialysis. Lane 1: total pellet (8M urea); Lane 2: Solubel fraction of the pellet (8M urea); Lane 3: Ni^{2+}–NTA flow-through; Lane 4: wash fraction 1; Lane 5: wash fraction 2; Lane 6: molecular weight marker; Line 7: elution fraction 1; Line 8: elution fraction 2; Line 9: elution fraction 3; Line 10: Supernatant of refolded PanLipΔN by stepwise dialysis. Proteins were resolved on a 12% SDS-PAGE gel and stained with Coomassie Brilliant Blue.

Table S1. Similarity of *Pseudonocardia antarctica* lipase against the accepted type proteins for each lipolytic family.
